# Impact assessment of the WHO FCTC over its first decade: methodology of the expert group

**DOI:** 10.1136/tobaccocontrol-2018-054374

**Published:** 2018-06-28

**Authors:** Geoffrey T Fong, Janet Chung-Hall, Lorraine Craig

**Affiliations:** 1 Department of Psychology, University of Waterloo, Waterloo, Ontario, Canada; 2 School of Public Health and Health Systems, Waterloo, Ontario, Canada; 3 Ontario Institute for Cancer Research, Toronto, Ontario, Canada

**Keywords:** WHO FCTC, impact assessment, research design

## Abstract

**Background:**

At its sixth meeting (Moscow, November 2014), the Conference of the Parties (COP) adopted decision FCTC/COP6(13) that called for an impact assessment to ‘examine the impact of the WHO Framework Convention on Tobacco Control (FCTC) on the implementation of tobacco control measures and on the effectiveness of its implementation’ after its first 10 years. An independent expert group (EG) was established to conduct the impact assessment, and report their findings at COP7 (Delhi, November 2016). This article describes the methodology used by the EG to conduct the first comprehensive multi-method assessment of the possible causal impact of the FCTC on global tobacco control over the past decade.

**Methods:**

The EG developed and followed a four-stage process model to conduct the impact assessment: (1) desk review of literature on FCTC impact; (2) collection and analysis of interview data from 12 country missions; (3) data synthesis and interpretation; and (4) preparation of a final report.

**Conclusions:**

The EG developed and engaged in a transparent and systematic process to conduct the FCTC impact assessment. The methods employed were rigorous, and explicitly guided by concerns about the difficulty of ascribing cause-and-effect relations. The EG’s report and supporting documents represent important sources of the positive impact of the Convention over its first decade. As development of the FCTC increasingly shifts to mechanisms for accelerating global implementation, the EG’s process model can be used as a methodology to assist Parties in carrying out their own assessments of the impact of the Treaty.

## Introduction

Smoking kills more than 7 million people worldwide annually,^1^ and ranks as the second largest contributor to global disability-adjusted life years.^2^ The FCTC was adopted in 2003 to address the global tobacco epidemic. Since its entry into force in 2005, the FCTC has become one of the most widely adopted United Nations treaties, with 181 Parties to date. Over the last decade, a growing number of countries have adopted various FCTC evidence-based tobacco control measures, and global smoking prevalence has decreased overall.^3^

The FCTC is considered to be an important catalyst for global tobacco control. Since the Convention came into force in 2005, the WHO Convention Secretariat has prepared seven reports that document global progress in the implementation of FCTC measures.^3–9^ These reports are based on the analysis of periodic reports on the implementation of the Convention submitted by Parties to the governing body of the FCTC, the COP, as called for under Article 21 (Reporting and exchange of information). Overall, these reports show steady progress in FCTC implementation, particularly for measures under Article 8 (Protection from exposure to tobacco smoke), Article 11 (Packaging and labelling of tobacco products), Article 16 (Sales to and by minors), and Article 12 (Education, communication, training and public awareness). These findings are comparable to those reported in the series of WHO reports on the status of the global tobacco epidemic.^10^ Consistent with the general downward trend in smoking prevalence observed in the Convention Secretariat’s 2016 report,^3^ two recent studies show that countries with strong implementation of core demand-reduction measures have experienced significant reductions in smoking prevalence over time.^11 12^ Together, these findings highlight the impact of the Convention on Parties’ implementation of tobacco control measures, and subsequent reductions in smoking prevalence. However, it is unclear to what extent advancements in tobacco control worldwide are potentially attributable to the FCTC.

The COP acknowledged the need for an overall assessment of the impact of the FCTC at its fifth meeting (Seoul, November 2012) under decision FCTC/COP5(12).^13^ At its sixth meeting (Moscow, November 2014), the COP adopted decision FCTC/COP6(13) that called for an impact assessment to ‘examine the impact of the WHO FCTC on the implementation of tobacco control measures and on the effectiveness of its implementation’ after its first 10 years of operation. As mandated by this decision, an independent expert group (EG) was established to conduct the impact assessment, and to report their findings to the COP at its seventh meeting (COP7; Delhi, November 2016).^14^ The work of the EG represents the first comprehensive multimethod assessment of the possible causal impact of the FCTC on developments in global tobacco control over the past decade.

This article describes the methodology used by the EG to conduct the FCTC impact assessment and compile a report of their findings for the COP. We provide an overview of the EG’s four-stage process model: desk review of existing scientific studies and other relevant literature on FCTC impact; collection and summary of interview data from 12 country missions; synthesis, interpretation and summary of all data sources; and report on the outcome of the impact assessment to COP7.

## Methods

### Selection of impact assessment EG

In April 2015, the Bureau of the COP selected a group of seven independent experts from nominations sent by FCTC Parties and non-governmental organisations (NGOs) that are observers to the COP (see table 1).

**Table 1:** Members of the Framework Convention on Tobacco Control impact assessment expert group

Expert group member	Affiliation
Pekka Puska (chair)	National Institute for Health and Welfare, Helsinki, Finland
Mike Daube (vice-chair)	Curtin University, Perth, Western Australia
Geoffrey T Fong (technical coordinator)	University of Waterloo, Waterloo, Ontario, Canada and Ontario Institute for Cancer Research, Toronto, Ontario, Canada
Sudhir Gupta	Directorate General of Health Services, Ministry of Health and Family Welfare, New Delhi, India
Thomas F McInerney	Treaty Effectiveness Initiative, Rome, Italy
Corne van Walbeek	University of Cape Town, Cape Town, South Africa
Ghazi Zaatari	American University of Beirut, Beirut, Lebanon

There were three meetings of the EG, all of which were convened at WHO Headquarters in Geneva, Switzerland. The EG defined the scope of the impact assessment exercise and developed a work plan at its first meeting (10–11 August 2015); reviewed progress and planned for additional work in the time period leading up to the COP7 meeting and created a draft outline for the EG report at a second meeting (26–28 January 2016); and discussed the content of their report to COP7 and dissemination of their findings at a third meeting (18–20 May 2016).

### Stages of the WHO FCTC impact assessment process

The EG’s evidence-gathering process focused on answering two overarching questions: (1) Did the FCTC increase and strengthen the implementation of tobacco control legislation? and (2) How effective are tobacco control measures, particularly those that align with the FCTC and its guidelines? The EG’s approach to the impact assessment was explicitly guided by concerns about the difficulty of ascribing cause-and-effect relations and therefore principles of causality were incorporated into all components.

The EG developed and followed a four-stage process model for FCTC impact assessment: (1) desk review of existing scientific studies and other relevant literature on FCTC impact; (2) collection and summary of interview data from 12 country missions; (3) synthesis, interpretation and summary of all data sources; and (4) preparation of a final report on the outcome of the impact assessment to COP7.

### Reviews of existing evidence on FCTC impact

Three desk reviews of the existing international literature on FCTC impact were commissioned by the Convention Secretariat^i^, as called for in decision FCTC/COP/6/13.^14^ These qualitative reviews served as a basis for the impact assessment and were made available to the EG at its first meeting in August 2015.

1. A global evidence review on the impact of the FCTC on the implementation of tobacco control legislation under 17 Articles of the Convention, and the effectiveness of those measures during the Treaty’s first decade, conducted by the International Tobacco Control Policy Evaluation Project (ITC Project).^15^ ^ii^
2. A report on the impact of the FCTC as a legal instrument to protect Parties’ tobacco control measures against domestic and international litigation and strengthen national tobacco control legislation, prepared in a collaboration between the McCabe Centre for Law and Cancer (a FCTC Knowledge Hub) and the International Legal Consortium of the Campaign for Tobacco-Free Kids.^16^
3. A report on tobacco industry responses to the FCTC, including strategies to block the implementation of measures called for under specific Articles, prepared by Stella Bialous, University of California, San Francisco.^17^

### Country missions

The COP6 decision mandated the EG to select 12 Parties, three from each of the four economic development levels prescribed by the World Bank, in consultation with the Bureau, for participation in site visits on a voluntary basis. The country missions were conducted as a qualitative process evaluation of the extent to which the FCTC contributed to the development and implementation of tobacco control measures, based on the perspectives of a broad range of key stakeholders in each country.

Three criteria were used for the selection of individual countries: (1) country must be an FCTC Party, (2) reliable surveillance data should preferably be available for analysis of prevalence and (3) reliable policy evaluation data should preferably be available for analysis of strength/effectiveness of FCTC implementation. In contrast to a needs assessment mission that focuses on a country’s record of tobacco control policy implementation, each impact assessment country mission was a process evaluation of whether and how the FCTC may have contributed to the development and implementation of tobacco control measures. It was thus essential that each of the 12 countries had some record of having implemented tobacco control measures.

In its first meeting, the EG recognised the importance of selecting the 12 countries to achieve an even distribution across the six WHO regions, while also meeting the requirements set forth in the COP6 decision to have three countries in each of the four World Bank income levels. Table 2 presents the 12 selected countries and mission dates.

**Table 2:** Selected countries for the Framework Convention on Tobacco Control impact assessment

Mission dates	Country	World Bank category	WHO region
30 November–2 December 2015	Kenya	Low-middle	AFR
17–19 January 2016	Iran (Islamic Republic of)	Upper-middle	EMR
19–21 January 2016	UK	High	EUR
21–24 February 2016	Madagascar	Low	AFR
23–26 February 2016	Turkey	Upper-middle	EUR
7–10 March 2016	Sri Lanka	Lower-middle	SEAR
28–31 March 2016	Republic of Korea	High	WPR
5–8 April 2016	Uruguay	High	AMR
12–15 April 2016	Philippines	Lower-middle	WPR
18–21 April 2016	Bangladesh	Low	SEAR
25–28 April 2016	Brazil	Upper-middle	AMR
2–5 May 2016	Pakistan	Lower-middle	EMR

AFR, African Region; AMR, Region of the Americas; EMR, Eastern Mediterranean Region; EUR, European Region; SEAR, South-East Asia Region; WPR, Western Pacific Region.

Prior to each country mission, each member of the EG that would be participating in the onsite visit was provided with country-specific briefing materials that summarised the status of FCTC implementation by Article (Articles 4–6 and 9–22). All country briefing materials were developed by the ITC Project in collaboration with the Convention Secretariat, based on information from peer-reviewed publications, grey literature, online databases (ie, Tobacco Control Laws, WHO FCTC implementation database) and consultations with FCTC focal points for tobacco control in each country.

During each country mission, semistructured interviews were conducted by 1–3 members of the EG, with assistance from one to two external consultants^iii^ provided by the Convention Secretariat. All interviews were conducted in English over a 3-day period. Key stakeholders in tobacco control were identified by FCTC focal points. The EG interviewed a broad range of stakeholders that were knowledgeable about FCTC implementation across the 12 countries: government representatives (n=217), parliamentarians (n=17), academics/researchers (n=25), civil society/NGO members (n=67), media (n=8) and WHO country/regional representatives (n=16) (see table 3).

**Table 3:** Number of tobacco control stakeholders interviewed during the 12 FCTC impact assessment country missions

Country	Government representatives	Parliamentarians	Academics/researchers	Civil society/NGOs	Media	WHO representatives
Kenya	25	4	–	12	2	2
Iran (Islamic Republic of)	17	–	4	–	–	–
UK	15	1	1	6	–	–
Madagascar	16	3	–	2	1	1
Turkey	24	–	4	6	–	1
Sri Lanka	17	8		5	2	2
Republic of Korea	11	–	5	10	–	–
Uruguay	16	1	2	4	–	1
Philippines	30	–	1	5	–	6
Bangladesh	12	–	5	9	3	–
Brazil	24	–	2	6	–	2
Pakistan	10	–	1	2	–	1
Total	217	17	25	67	8	16

FCTC, Framework Convention on Tobacco Control; NGO, non-governmental organisation.

The EG used three criteria to develop an interview guide of open-ended questions for assessment of the possible causal relationship between FCTC and policy action in each of the FCTC policy domains (online supplementary file 1).

10.1136/tobaccocontrol-2018-054374.supp1 Supplementary file 1

#### Criterion 1: Temporal precedence—Did the FCTC precede action in the country in a particular policy domain?

To determine the temporality of the FCTC as the putative cause for action in each policy domain, timing of policy implementations was considered in relation to three key dates in the FCTC implementation timeline: FCTC adoption in May 2003, FCTC entry into force in February 2005 and the date each country of interest ratified the Treaty. In order to account for different policy timelines, questions on the temporality of the FCTC were asked in each policy domain. Policy milestones that were considered as indicators of governmental action included: consideration of legislation and formal actions (eg, draft bill, internal report calling for policy action, first reading of and/or passage of draft legislation, establishment of regulations under the authority of an existing act, implementation of law/regulations).

#### Criterion 2: Covariation between cause and effect—Is there covariation between the FCTC and action in the country in a particular policy domain? 

Covariation between cause and effect was considered across the 12 countries.

#### Criterion 3: Internal validity assessment of the possible causal relationship—Is there evidence that the FCTC caused the governmental action in this policy domain?

Counterfactual questions that explicitly mentioned the focus of the process evaluation were used in all interviews to reduce possible ‘positive response bias’ that focuses on highlighting a country’s achievements in tobacco control and ‘negative response bias’ that criticises the FCTC and/or lack of progress in tobacco control. These counterfactual questions included, ‘Would your country have implemented [tobacco control policy] if there was no FCTC?’ ‘Would your country have developed [tobacco control policy]/would it have been taken up in Parliament/would it have Passed/would it have been implemented…if there was no FCTC?’ Follow-up questions were then used to probe for value-added contributions of the WHO FCTC: ‘If there was no FCTC, would this same governmental action have occurred? If so, would it have happened as quickly? And would the action have been as strong?’ The focus of the EG’s inquiries was not on the level of accomplishments of the country’s tobacco control measures, but rather on whether the FCTC had helped in the development of measures, increased their strength, accelerated their development, strengthened political will for measures and used to counter opposition to measures. The distinction between focusing on outcomes and focusing on the FCTC’s contributions was appreciated and understood by both supporters of government and by critics.

All interviews were audio-taped using a digital voice recorder. The ITC Project transcribed digital recordings verbatim. Interview transcripts, field notes from members of the EG and external consultant reports from each country visit were then synthesised by ITC Project researchers into country mission reports ranging in length from 15 to 25 pages.^iv^ Each country mission report summarised the positive outcomes and challenges in WHO FCTC implementation, with representative quotes included to support central themes. All country mission reports were made available to the EG prior to its third and final meeting in May 2016.

### Feedback from WHO regional advisors

The Convention Secretariat developed a set of 14 open-ended survey questions for assessment of the regional impact of the FCTC. In February 2016, the surveys were sent to regional advisors for tobacco control in each of the six WHO regions. Findings based on the collective responses submitted by the WHO regional advisors were then summarised in a report that was made available to the EG in April 2016.

### Synthesis, interpretation and summary of evidence

A draft outline for the EG’s impact assessment report was prepared at its second meeting in January 2016, and an initial draft of the report was created at its third meeting in May 2016. To support the EG in drafting their report, the ITC Project prepared summaries of evidence from the country mission reports and desk reviews for each section of the report, and main themes on positive outcomes and challenges in WHO FCTC implementation based on findings from country missions.

In addition to these evidence reviews and country mission reports, new quantitative analyses were conducted by Gravely *et al* 12 across 107 Parties to examine the association between implementation of key FCTC demand-reduction measures between 2008 and 2014 and changes in smoking prevalence between 2005 and 2015, the first decade of the Convention. These analyses demonstrated that Parties that had implemented a greater number of the five demand-reduction measures (Articles 6, 8, 11, 12, 14) at the highest level (as coded by the WHO, and corresponding approximately to having fully met the elaborated guidelines for that FCTC Article) experienced significant reductions in smoking prevalence across the first decade of the FCTC. The results of these analyses were made available to the EG.

The EG’s final report was submitted to the COP on 31 May 2016, and the EG’s report and findings were presented during a plenary session and at a briefing during the COP on 7 November 2016. The EG’s final report and supplementary documents, including the international evidence review, the two external reports and other relevant material, were posted on the Secretariat website (http://www.who.int/fctc/cop/cop7/Documentation-Supplementary-information/en/).

**Figure 1:**
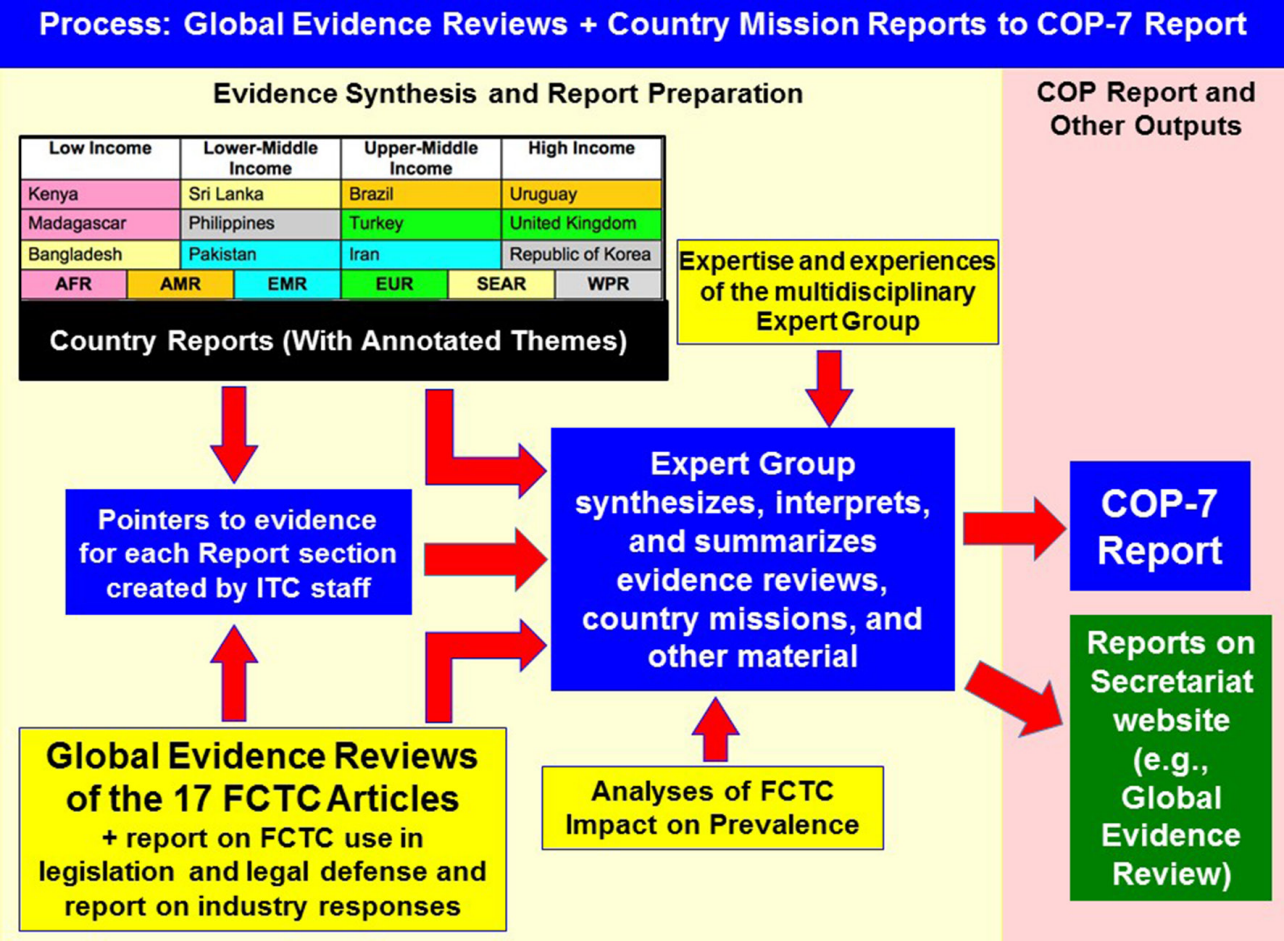
FCTC impact assessment expert group process model. AFR, African Region; AMR, Region of the Americas; COP, Conference of the Parties; EMR, Eastern Mediterranean Region; EUR, European Region; FCTC, Framework Convention on Tobacco Control; ITC, International Tobacco Control Policy Evaluation; SEAR, South-East Asia Region; WPR, Western Pacific Region.

Figure 1 summarises the EG’s process of integrating the evidence sources in preparing their final report on the findings of the impact assessment.

## Conclusions

The FCTC impact assessment carried out by the EG, as mandated in decision FCTC/COP6(13), is the first comprehensive multimethod assessment of the impact of the FCTC on developments in global tobacco control over its first decade of operation. The EG developed and engaged in a transparent and systematic process to conduct the impact assessment. Rigorous methods were used by the EG to gather evidence through desk reviews, country missions and engagement with WHO regional offices; analyse all evidence sources; and create a report of their findings for COP7. The use of a multimethod approach for conducting the impact assessment was important given the broad scope of the EG’s objective to assess FCTC impact over its first decade. Moreover, using multiple components allowed the EG to capitalise on the individual strengths of each method while offsetting their limitations and biases, thereby increasing the confidence in the validity of convergent findings. The EG’s procedures were explicitly guided by concerns about the difficulty of ascribing cause-and-effect relations, and informed by guidelines that provided key suggestions for interview questions aiming to assess whether the FCTC was indeed a causal factor in the implementation of tobacco control measures and in the effectiveness of those measures.

The EG’s report, together with the other reports and documents prepared as evidence for the EG, provide comprehensive documentation of the positive impact of the FCTC over its first decade and remaining obstacles. As the focus of the COP has shifted from building the FCTC through ratifications and the development of guidelines to accelerating and strengthening implementation of the FCTC, the EG’s process model can provide a methodology to support Parties in carrying out their own FCTC impact assessments.

**What this paper adds**

- The Framework Convention on Tobacco Control (FCTC) Impact Assessment conducted by the expert group (EG; created by a decision of the Conference of the Parties (COP) at its sixth meeting (Moscow, October 2014) (FCTC/COP6(13)) is the first comprehensive multimethod evaluation of the impact of the FCTC over the Treaty’s first decade.
- This paper describes the methodology that the EG used to gather and analyse evidence relating to the impact of the WHO FCTC across 17 Articles of the Treaty and the procedures they used to prepare a report of their findings to the COP at its seventh meeting (Delhi, November 2016).
- The EG’s rigorous and systematic methods can be used to guide Parties who may wish to conduct their own impact assessments, facilitate evidence-based policy-making and advance implementation of the Convention.

## Footnotes

i Given the limited timeframe for the preparation of these materials, these were not systematic reviews of existing literature.

ii Partial funding was also provided by Health Canada.

iii External consultants: Paula Beltran, Daniel Ferrante, Trinette Lee and Patrick Musavuli.

iv Due to technical difficulties, audio-recordings of interviews conducted in the Republic of Korea were not available. For this reason, the International Tobacco Control Policy Evaluation Project did not prepare a country mission report for the Republic of Korea.
