# Supplementary material for: Impact assessment of the WHO FCTC over its first decade: methodology of the expert group
Source: Tob Control. 2018 Jun 28;28(Suppl 2):s84–8. doi: 10.1136/tobaccocontrol-2018-054374 (PMC6589466; doi:10.1136/tobaccocontrol-2018-054374)
Supplement: Supplementary file 1 [file tobaccocontrol-2018-054374supp001.pdf]

Supplementary file 1. WHO FCTC Impact Assessment: analysis of purpose and suggestions for cross-cutting WHO FCTC impact questions, prepared by Geoffrey T. Fong for the first meeting of the WHO FCTC impact assessment expert group (Geneva, Switzerland, 10-11 August 2015)

According to Decision FCTC/COP6(13)—Impact Assessment…

"(2) …the purpose of the impact assessment should be to assess and examine the impact of the WHO FCTC on implementation of tobacco control measures and on the effectiveness of its implementation in order to assess the impact of the Convention as a tool for reducing tobacco consumption and prevalence after its first 10 years of operation."

To assess impact of the FCTC on implementation and on effectiveness of implementation, it is necessary to consider the evidence relating to the three necessary conditions for causality:

1. **Temporal Precedence: Did the FCTC precede action in the country in a particular policy domain?**
   - **a.** Temporal precedence is a requirement for causality, but the situation for our Expert Group requires a more complex set of considerations than in physics (to be sure), and even in program evaluation, where a specific timepoint of the program is often known:
     - **i.** Temporality of the putative cause—the FCTC. Which is the relevant aspect of the FCTC that should be used as the timepoint: adoption of the FCTC in May 2003? When the FCTC came into force for the world in February 2005? When the specific country of interest became an FCTC Party? All of these are possible dates of relevance in determining the temporality of the FCTC as the putative cause.
       - **1.** Temporality of the FCTC in EACH policy domain: FCTC Article Guidelines are the most direct and specific articulation of the FCTC in its implementation for each Article, and thus it is important to consider the timepoint of the Article Guidelines. Analysis of whether the FCTC caused action must be conducted article-by-article because each domain will have its own timeline (e.g., tax increases may well occur at different timepoints than a smoke-free law, which will also differ from the timepoint of the implementation of pictorial warnings).
     - **ii.** Temporality of the effects—governmental action in this particular domain: Which is the relevant milestone: when the Act/Bill/Regulation/Measure was first being considered? When concrete action was taken within the government (e.g., draft bill? Internal report suggesting action in this policy domain?) First reading? Passage of the bill? Establishment of regulations (in those countries where there exists a separation between bill/act and regulations)? Implementation of the law/regulations?
       - **1.** Again, the temporality of the effects will vary across different FCTC articles as stated above.

2. **Covariation between cause and effect: Is there covariation between the FCTC and action in the country in a particular policy domain?**
    a. This requirement of covariation between cause and effect is not really relevant to us WITHIN a country because we will not have multiple events within a single country that will allow us to make any inferences based on covariation of cause and effect. However, it is the case that ACROSS the 12 countries, the covariation will be meaningful. For example, if it is the case that in 9 out of 12 countries, there was reasonable covariation between FCTC and action within a given policy domain—for example, if smoke-free laws were passed and implemented AFTER the FCTC (and there exists evidence of the influence of FCTC to smoke-free laws from government officials—see next point—whereas in 3 countries there was not any evidence for a link between FCTC and smoke-free laws (e.g., they happened before the FCTC), then the 9/12 represents evidence in the "positive-positive" cell of the 2 x 2 matrix of cause and effect, and that at least is evidence consistent with covariation between FCTC and smoke-free laws. Not perfect, but the best we can do, I think, given the nature of the task and the limitations on sample size (N=12).

3. **Internal validity assessment of the possible causal relationship: Is there evidence that the FCTC caused the governmental action in this policy domain?**
    — Here, my analysis is tailored to the task we face (slightly different from the textbook treatment of this issue). There are TWO criteria, of which the first is more important than the second:
    a. **Is there *convergent* evidence that the FCTC CAUSED ("led to", "influenced", and similar causal/quasi-causal terminology) governmental action in this policy domain?**
        i. Do government officials/key informants report that the FCTC was influential in formulating the policy action: Motivating its action at all? Accelerating the action that was already planned? Shaping its content (e.g., including, or modifying (strengthening) key provisions that were there already, for example, broadening the set of public venues covered by smoke-free legislation, or strengthening enforcement in ways consistent with Article 8 Guidelines)?
        ii. This would be considered **positive** evidence for the causal relationship.
        iii. Another source of evidence besides key informant reports is the text of the law/regulation itself: if it uses phrases from the Guidelines or the treaty text, then that should be considered **positive** evidence for the causal relationship.
    b. **Is there *divergent* evidence that OTHER possible causes for governmental action in this policy domain are not viable?**
        i. This criterion is typically stated in textbooks, because it is important to demonstrate that other possible causes could not account for the effect. I only list this here for completion, but also to comment that this criterion is not important for us. We would not ever claim that other possible causes did not also contribute to positive policy action. After all, any policy decisions are

ultimately those that work through the political process, and thus there are definitely those contributing causes (e.g., action consistent with already an existing political agenda) that will be at work here.

c. **So a strong case can be made for the criterion for causality = demonstrating that the FCTC was AMONG the causes that contributed to governmental action. It is of course NOT necessary to demonstrate that the FCTC was the ONLY cause.**

With the above criterion in mind, the following set of questions could be asked to gain information on the possible **causal** relationship between FCTC and policy action in **each of the policy domains of the FCTC.**

General Questions about integration of tobacco control measures with other/broader initiatives and other sectoral plans and the FCTC's role in that integration

- **Linkage to UNDP, UNDAF, NCD**
- **Questions on Article 5 to be added for list of questions at the beginning of the impact assessment.**

We envision asking these questions for each of the articles of the FCTC:

Prelude: indicate that the legislation/regulations/policies we are referring to will include any action taken or considered relevant to all smoked tobacco products (e.g., cigarettes, waterpipe, bidi) and smokeless tobacco products, and alternative nicotine delivery products (e.g., ENDS electronic cigarettes)

1. What (identify policy domain) legislation/regulations/policies were implemented before your country ratified the FCTC?
2. What legislation/regulations/policies were implemented after the Party ratified the FCTC?
3. What resources (e.g., budget, personnel) if any were made available for the implementation of this/these legislation/regulations/policies?
4. What level of influence did the FCTC have on the policy development and implementation process:
    a. The FCTC helped to guide or define the scope and content of legislation/regulations/policies.
    b. The FCTC helped to build the support needed for passing the legislation/regulations/policies.
    c. Other
5. Who used the FCTC in the legislative and regulatory processes of this policy domain and how did they use it?
6. What are/were the main challenges/barriers that you faced/are facing in the formulation and enactment of legislation/regulations/policies in this policy domain?
7. Do you think your government would have implemented (identify policy domain) legislation/regulations/policies if your country was NOT a Party to the FCTC? Why or why not?
8. Were the FCTC Guidelines (if relevant) used in the formulation and implementation of those legislation/regulations/policies? If yes, how were the Guidelines used?

9. What are your specific priorities within (this policy domain)?
10. In formulation of legislation/regulations/policies in this policy domain, to what extent were specific population groups (e.g., women, children, low-socioeconomic groups) considered?
11. What are/were the main challenges/barriers that you faced/are facing in strengthening the effectiveness of the legislation/regulations/policies in this policy domain?
12. Is your country working towards further action (in this policy domain) to strengthen your country's implementation of Article x? (e.g., new legislation or stronger enforcement) If yes, please describe.
13. Are you aware of any studies in your country that have been conducted to evaluate the impact of (identify policy domain) legislation/regulations/policies implemented after the FCTC was ratified? Can you provide the details of these studies including:
    a. who conducted the study(ies)
    b. what were the findings
    c. how have the findings been used
    d. copies/links of any resulting reports/publications on the findings
       NOTE: this request for studies will be sent to the Party in advance of the visit.
14. Are you aware of any ongoing studies or research in your country to monitor and evaluate whether existing (identify policy domain) legislation/regulations/policies are effective? If yes, please provide details.
15. Does your country have dedicated government or non-government funding for studies to evaluate the impact of (identify policy domain) legislation/regulations/policies?
